# Estimating the Number of Vulnerable People in the United States Exposed to Residential Wood Smoke: Rogalsky et al. Respond

**DOI:** 10.1289/ehp.1409136R

**Published:** 2015-02-01

**Authors:** Derek K. Rogalsky, Pauline Mendola, Tricia A. Metts, William J. Martin

**Affiliations:** 1 Georgetown University School of Medicine, Georgetown University, Washington, DC, USA; 2 *Eunice Kennedy Shriver* National Institute of Child Health and Human Development, National Institutes of Health, Department of Health and Human Services, Bethesda, Maryland, USA; 3 Department of Environmental Health, East Tennessee State University, Johnson City, Tennessee, USA

We appreciate the thoughtful comments by Noonan et al. and consider them an important contribution to the dialogue surrounding the public health issue of residential wood smoke. Their comments accurately reflect and deal fairly with the issues raised in our recent article, “Estimating the Number of Low-Income Americans Exposed to Household Air Pollution from Burning Solid Fuels” (Rogalsky et al. 2014). Absent the availability of new data on household exposure to residential wood smoke in the United States, we agree that it is challenging to develop an accurate estimate of the potential risk. It is true that we were purposefully conservative in our assumptions in order to estimate the household air pollution (HAP) burden among the most vulnerable Americans. We appreciate the alternate estimate that Noonan et al. have offered, although we would argue that their estimate likely includes a significant number of individuals with only intermittent exposure to high levels of HAP.

Our estimate began with the 2.8 million households that use wood, coal, or coke as the primary means of household heating, but we also recognize that an additional 8.8 million homes use wood as a secondary heating source. Nearly 5 million households from these two groups use a wood-burning stove, whereas the remainder typically use fireplaces. We believe that it is reasonable to assume that primary users heat their homes daily with wood during the heating season, but this assumption does not hold for secondary users. An unknown but significant number of these households may heat their homes with wood only on rare occasions, resulting in less frequent HAP exposure.

Noonan et al. excluded homes with wood-burning fireplaces in their estimate because the frequency of use in this group is unknown, but they did include secondary users of wood stoves for whom there are also no data. Combining all secondary users of a wood stove into the same group as primary users likely includes many homes with infrequent or episodic HAP exposure.

Noonan et al. also note that we were conservative in our estimate by limiting the at-risk households to those below the federal poverty level. Children and the elderly residing in homes that burn wood may certainly reflect an at-risk population regardless of socioeconomic status, and this notion does merit consideration. However, by not focusing on poverty, Noonan et al. bring into their estimate all wood stove users, including the most affluent users most likely to have clean-burning stoves with regular maintenance. This is an important point for discussion and further research because—as is clear from previous work on this topic, much of it done by Noonan, Ward, and colleagues—even in communities with a relatively high proportion of the population below the federal poverty level, only 53–65% of homes exceed the World Health Organization standard for particulate matter of < 2.5 µm/24 hr (Noonan et al. 2012; World Health Organization 2005). It is unclear, given the current published data on the subject, whether this proportion of homes would be different in more affluent communities, but it seems likely, given the data from international sources that strongly link poverty and HAP from burning solid fuels (World Bank 2011).

Noonan et al. are correct in pointing out the limitations inherent in our estimates. Our estimates did not account for the regional and neighborhood effects of wood smoke. This is relevant because communities with a high proportion of wood-burning homes experience significant infiltration of outdoor air pollution. Unfortunately, from an estimation standpoint, these issues are extremely variable by region and weather patterns and are not easily incorporated into national estimates such as ours. However, it bears repeating that the negative health effects of HAP have been demonstrated most consistently in those with daily, chronic exposure to high concentrations of particulate matter; therefore, it seems likely that frequent direct exposure would correlate most closely with the negative health impacts (World Bank 2011).

Ultimately, in our research, we and Noonan et al. struggle with a lack of available data on exposure to HAP in U.S.-based households. Going forward, we recommend that future studies include measures such as household, personal, and ambient air monitoring to determine whether levels are consistent with impairment of human health and how frequently these levels are attained in such households. We also recommend that the U.S. Census Bureau should, in the next installment of the American Community Survey, ask respondents to quantify how frequently they use their wood-burning appliances or fireplaces to heat their homes as either a primary or secondary source of heating. This information would allow researchers to better understand the overall number of Americans potentially at risk.

HAP is an environmental justice issue of clear public health importance, both globally and in its disproportionate impact on mostly rural Americans. We wholeheartedly join Noonan et al. in advocating for those affected.
